# Quality improvement in the golden hour for premature infants: a scoping review

**DOI:** 10.1186/s12887-024-04558-9

**Published:** 2024-02-01

**Authors:** Lijuan Sheng, Guichao Zhong, Ruirui Xing, Xudong Yan, Huanjin Cui, Zhangbin Yu

**Affiliations:** Department of Neonatology, Shenzhen People’s Hospital, (The Second Clinical Medical College, Jinan University;The First Affiliated Hospital, Southern University of Science and Technology), Shenzhen, 518020 Guangdong China

**Keywords:** Quality improvement, Golden hour, Extremely premature infants, Extremely low birth weight infants, Scoping review

## Abstract

**Background and objective:**

Evidence-based research has shown that golden hour quality improvement (QI) measures can improve the quality of care and reduce serious complications of premature infants. Herein, we sought to review golden hour QI studies to evaluate the impact on the outcome of preterm infants.

**Methods:**

A comprehensive literature search was conducted in PubMed, Embase, Cochrane Library, and SinoMed databases from inception to April 03, 2023. Only studies describing QI interventions in the golden hour of preterm infants were included. Outcomes were summarized and qualitative synthesis was performed.

**Results:**

Ten studies were eligible for inclusion. All studies were from single centers, of which nine were conducted in the USA and one in Israel. Seven were pre-post comparative studies and three were observational studies. Most included studies were of medium quality (80%). The most common primary outcome was admission temperatures and glucose. Five studies (*n* = 2308) reported improvements in the admission temperature and three studies (*n* = 2052) reported improvements in hypoglycemia after QI. Four studies (*n* = 907) showed that the incidence of bronchopulmonary dysplasia (BPD) was lower in preterm infants after QI: 106/408 (26.0%) vs. 122/424(29.5%) [OR = 0.68, 95% CI 0.48–0.97, *p* = 0.04].

**Conclusions:**

Our study showed that the golden hour QI bundle can improve the short-term and long-term outcomes for extremely preterm infants. There was considerable heterogeneity and deficiencies in the included studies, and the variation in impact on outcomes suggests the need to use standardized and validated measures. Future studies are needed to develop locally appropriate, high-quality, and replicable QI projects.

**Supplementary Information:**

The online version contains supplementary material available at 10.1186/s12887-024-04558-9.

## Introduction

Premature infants are a very vulnerable patient group, and prematurity is the leading cause of neonatal death worldwide. The mortality rate of extremely preterm infants (less than 27 weeks gestation) and extremely low birth weight (ELBW) infants (< 1000 g) is 30–50% [1]. Survivors are also at risk of BPD, necrotizing enterocolitis (NEC), intraventricular hemorrhage (IVH), retinopathy of prematurity (ROP), and septicemia [1].

The “golden hour” is a term initially used in adult trauma emergency care; however, it has increasingly been applied to other medical fields, including neonatology, with a particular focus on extremely low gestational age (GA) preterm infants [2, 3]. For preterm infants, the “golden hour” is the first hour of life after delivery and includes the time during resuscitation in the delivery room (DR), transport and admission to the neonatal intensive care unit (NICU). It involves a series of interdependent tasks and procedures of DR resuscitation and maintenance of stability during NICU admission [3].

The importance of the initial treatment after birth of preterm infants has been demonstrated in previous studies [4, 5]. Studies have found that hypothermia is prevalent in very low birth weight (VLBW) infants and is associated with IVH and mortality [6, 7]. Several studies demonstrated that quality improvement (QI) improved admission hypothermia in preterm infants [8–11]. Other studies on the golden hour interventions for premature infants have focused on outcomes including BPD, IVH, or hypotension [12–16]. Despite the complexity of the early care, golden hour QI protocols including evidence-based practices and standardized implementation process to the first few minutes after birth have been used to improve the quality of care for extremely premature and extremely low birth weight (EP-ELBW) infants [17–22].

A synthesis of literature can provide valuable information for clinical decision-making and future research. However, to the best of our knowledge, no study has systematically summarized the existing literature on the implementation of golden hour QI protocol and evaluated its impact on the outcomes of premature infants. Therefore, we sought to conduct a scoping review to summarize these studies. We chose the scoping review methodology, because it can be conducted to meet various objectives, allowed the authors to include multiple study outcomes and explore a broad clinical question [23].

## Methods

This scoping review followed the Preferred Reporting Items for Systematic reviews and Meta-Analyses extension for Scoping Reviews (PRISMA-ScR) [23].

### Search strategy

A comprehensive literature search was conducted in PubMed, Embase, the Cochrane Library, and SinoMed databases from inception to April 03, 2023. Relevant studies were identified and search accuracy was maximized using the following terms: golden hour, QI, preterm infants, and newborn. There were no search limits or restrictions. Moreover, reference lists of included studies and relevant systematic reviews were further searched to identify additional studies. The detailed search strategy in PubMed is shown in Supplementary material 1 in the addendum.

### Eligibility criteria

Articles were eligible for inclusion if: (1) the population was preterm infants, very-low-birth-weight (VLBW) infants, or extremely-low-birth-weight (ELBW) infants; (2) the intervention was improvement initiatives focused on the golden hour after birth and presented outcomes over time; (3) eligible study designs were randomized controlled trials (RCTs), non-randomized interventional studies, pre-post comparative studies, or interrupted time series study (ITS). Articles were excluded if they investigated only a single intervention. Conference abstracts and review articles were also excluded. There was no limitation on the publication language.

### Outcome measures

The primary outcomes included admission temperatures, serum glucose concentration, time to completion of stabilization. Hypoglycemia was defined as glucose < 45 mg/dL. Time to completion of stabilization was defined as time to close of incubator top in minutes. Completion of admission stabilization included DR resuscitation, initiation of intravenous fluids and antibiotics (when indicated), the establishment of central access, initiation of humidity, and provision of decreased environmental stimulation to approximate the intrauterine environment, and closure of the top of the isolette.

Process outcomes included time to intubation, time to surfactant, time to NICU admission, time to admission temperature, time to umbilical line placement(confirmation), time to initiation of IV fluids, time to initiation of antibiotics. All times were defined as the time from birth to measurement in minutes.

Balancing outcomes included hyperthermia, insertion-related catheter-associated bloodstream infections, irrational use of antibiotics (defined as antibiotics initiated without a physician order), mechanical ventilation duration, and postmenstrual age (PMA) on the day of discharge home.

The main long-term outcomes included: severe IVH (Grade III or IV hemorrhages), BPD (defined as oxygen or positive pressure support required at 36 weeks PMA), and mortality.

### Study selection and data extraction

Two researchers (LJS and GCZ) independently screened the titles, abstracts, and full texts of all studies for eligibility. Any discrepancies were resolved through a consensus discussion with a third author. Two researchers (LJS and RRX) independently extracted data using a standardized data collection form. The following details were extracted from each study: author(s), publication year, location, setting, study duration, sample size, target population, study design, QI bundled elements, primary outcomes, secondary outcomes, process outcomes and balancing outcomes. Any differences in the abstracted data were resolved by consensus with a third researcher (ZBY).

### Critical appraisal

The Quality Improvement Minimum Quality Criteria Set (QI-MQCS) checklist was used for the critical appraisal of the methodological quality of the included studies [23]. It includes the following 16 content domains: Organizational Motivation, Intervention Rationale, Intervention Description, Organizational Characteristics, Implementation, Study Design, Comparator, Data Source, Timing, Adherence/Fidelity, Health Outcomes, Organizational Readiness, Penetration/Reach, Sustainability, Spread, and Limitations. Each study was evaluated based on the 16 content domains, with each domain recording 1 point if it met the minimum criteria, and 0 points if it did not. Based on the criteria, the included studies were rated as high, medium, and low quality, where > 10 indicated high quality, 7–10 indicated medium quality, and < 7 indicated low quality. Finally, two authors (LJS. and GCZ) independently assessed the studies, and discrepancies were resolved by group discussion.

### Data analysis

Golden hour QI bundled elements were summarized as frequencies and percentages. The histogram of the detailed interventions of the Golden Hour bundled items was constructed in Microsoft Excel 2009. The meta-analysis was conducted using Review Manager 5.4 software. Due to the absence of heterogeneity between studies (I^2^ ≤ 50%), a fixed-effects model approach was used to analysis. We reported RR and 95% CI using the Mantel–Haenszel (MH) method for dichotomous outcomes to summarize the effect on long-term outcomes. *P* < 0.05 was considered significant.

## Results

**Fig. 1:**
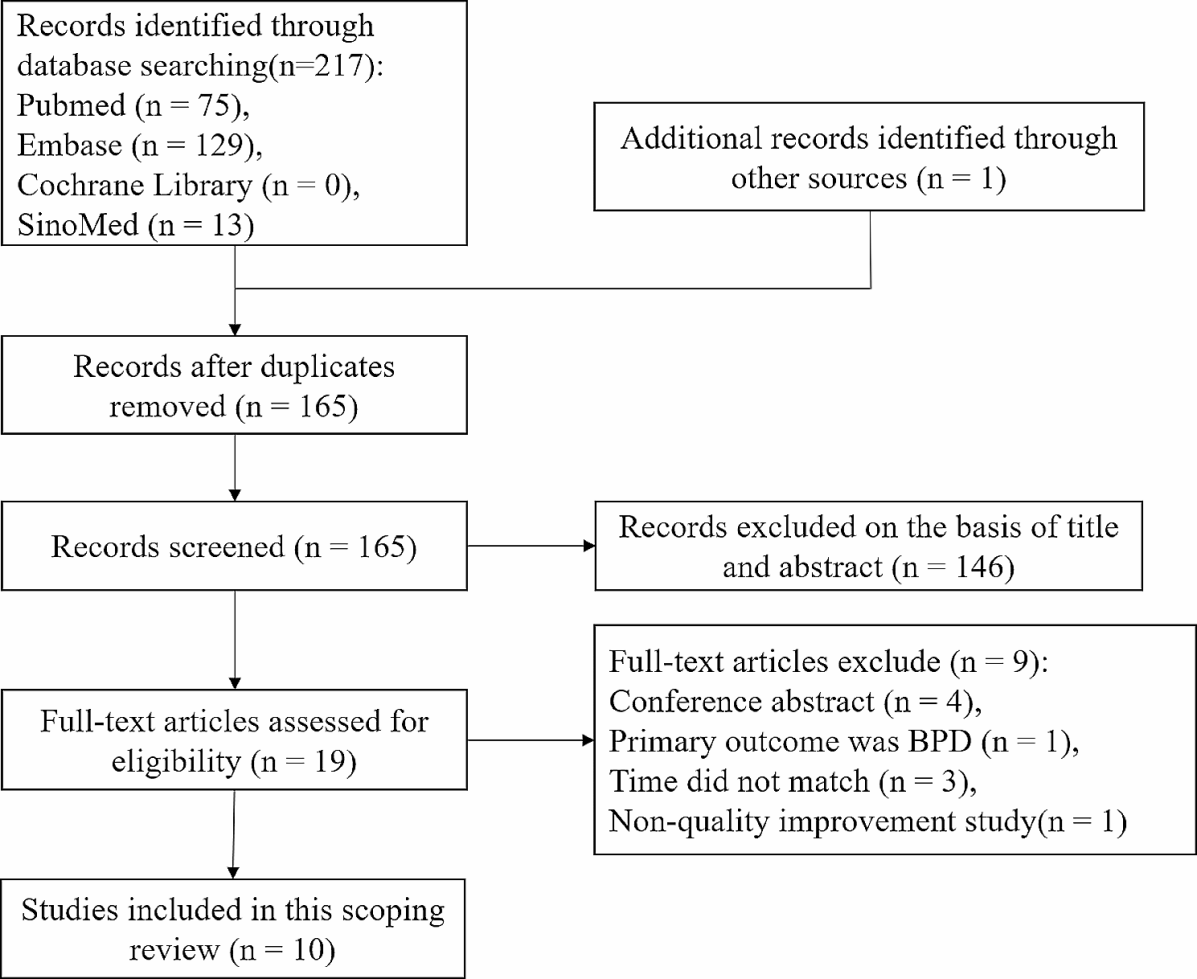
Study selection flow chart

A total of 218 articles were initially identified, and 53 duplicates were excluded. Of the remaining 165 articles, 146 completely unrelated studies were excluded after screening titles and abstracts. After a full-text review of the remaining 19 studies, nine studies were excluded for the following reasons: three studies were excluded because they did not include a complete intervention in the first hour after birth [12, 24, 25], one study was excluded due to a mismatch in primary outcome [13], four studies were conference abstracts [26–29], and one study was not QI research [30]. Ten studies were finally included in the scoping review [17–21, 31–35]. The study selection flow chart is shown in Fig. 1.

### Study characteristics

Characteristics of the included studies are detailed in Table 1. Nine studies were conducted in the USA and one study was conducted in Israel. All studies were from single centers. Seven reports were pre-post comparative studies (70%) and three were observational studies. The studies were published between 1999 and 2017. The sample sizes ranged from 24 to 1439 (median = 190). Six studies used GA alone as an inclusion criterion and three studies used GA and BW as an inclusion criterion; GA ranged from < 27 weeks to < 33 weeks and BW ranged from < 1000 g to < 1500 g. One report used BW (< 1500 g) alone as an inclusion criterion.

**Table 1:** Characteristic of included studies

References	Duration	Country	Size	GA/BW	Study design	Primary outcomes	Long-term outcomes
Croop et al. [32]	2012.01-2017.03	USA	294	<27week	Retrospective-prospective cohort	Admission temperatures, Serum glucose concentration, Time to completion of stabilization	Severe IVH, BPD, ROP requiring treatment
Peleg et al. [17]	2015.09-2017.03	Israel	388	≤ 32^+ 6^week	Retrospective case- control	Admission temperatures, Serum glucose concentration	Severe IVH, BPD, LOS, NEC, mortality
Harriman et al. [33]	NR (9month)	USA	24	≤ 32week	Before/after	Admission temperatures, Time to completion of stabilization	NR
Ashmeade et al. [18]	2007.12-2011.06	USA	295	≤ 28week or ≤ 1000 g	Before/after	Admission temperatures, Serum glucose concentration	Severe IVH, BPD, ROP requiring treatment, LOS, NEC, mortality
Lambeth et al. [34]	2013.05-2014.08	USA	155	<1500 g	Before/after	Admission temperatures, Serum glucose concentration	NR
Vergales et al. [21]	2008.06-2012.12	USA	152	<27week	Before/after	Admission temperatures	Severe IVH, BPD, mortality
Reuter et al. [19]	2011.07-2013.07	USA	72	≤ 29week or <1000 g	Retrospective cohort	Admission temperatures	Severe IVH, BPD, NEC, PDA, mortality
Castrodale et al. [20]	2008.05-2011.12	USA	225	<28week	Retrospective cohort	Admission temperatures, Serum glucose concentration	NR
Wallingford et al. [31]	2011.03-2011.09	USA	49	<33week	Before/after	Admission temperatures, Time to completion of stabilization	BPD
Reynolds et al. [35]	1999.01-2008.01	USA	1439	<32week or<1500 g	Before/after	Admission temperatures	BPD, ROP requiring treatment

GA: gestational age, BW: birth weight, NR: not reported

BPD: bronchopulmonary dysplasia; NEC: Necrotizing enterocolitis; PMA: postmenstrual age; IVH: intraventricular hemorrhage; ROP: retinopathy of prematurity; LOS: late-onset sepsis; PDA: patent ductus arteriosus

### Critical appraisal

QI-MQCS was used to assess QI publications, and all included studies had scores between 7 and 14 points (Table 2). Eight studies were medium-quality while two were high-quality. All studies met the minimum quality criteria for five of the 16 domains, including Organizational Motivation, Intervention Rationale, Intervention Description, Implementation and Organizational readiness. Most of the studies, however, failed to meet the minimum standards in various areas, including the lack of information about comparator care processes (Comparator, 6/10), data sources and outcome definition (Data source, 5/10), compliance with the intervention for the duration of the study, fidelity data on intervention use, or described mechanisms that ensure compliance (Adherence/Fidelity, 7/10), the number of units or sites participating in the intervention compared to the available/eligible units (Penetration/Reach, 10/10), the sustainability or the potential for sustainability (Sustainability, 9/10), and the potential for spread, existing tools for spread, or spread attempts/large-scale rollout (Spread, 9/10).

**Table 2:** Critical Appraisal of studies using QI-MQCS [23]

References	(1)	(2)	(3)	(4)	(5)	(6)	(7)	(8)	(9)	(10)	(11)	(12)	(13)	(14)	(15)	(16)	Total score
Croop et al. [32]	1	1	1	1	1	1	1	1	1	1	1	1	0	1	0	1	14
Peleg et al. [17]	1	1	1	1	1	1	1	0	0	0	1	1	0	0	0	1	10
Harriman et al. [33]	1	1	1	1	1	1	1	1	0	0	0	1	0	0	0	1	10
Ashmeade et al. [18]	1	1	1	1	1	0	0	1	1	0	1	1	0	0	0	1	10
Lambeth et al. [34]	1	1	1	1	1	0	1	1	1	1	1	1	0	0	0	1	12
Vergales et al. [21]	1	1	1	1	1	1	0	0	1	0	1	1	0	0	0	1	10
Reuter et al. [19]	1	1	1	1	1	1	0	0	1	0	1	1	0	0	1	0	10
Castrodale et al. [20]	1	1	1	1	1	1	0	1	1	0	0	1	0	0	0	0	9
Wallingford et al. [31]	1	1	1	0	1	0	0	0	0	1	1	1	0	0	0	0	7
Reynolds et al. [35]	1	1	1	1	1	0	0	0	1	0	1	1	0	0	0	0	8

(1) Organizational motivation, (2) intervention rationale, (3) intervention description, (4) organizational characteristics, (5) implementation, (6) study design, (7) comparator, (8) data source, (9) timing, (10) adherence/fidelity, (11) health outcomes, (12) organizational readiness, (13) penetration/reach, (14) sustainability, (15) spread, (16) limitations

### QI bundled elements

QI bundle elements included 11 interventions (Table 3). The most common elements were antenatal counseling and team briefing (10/10), prevention of hypothermia (10/10), respiratory system support (10/10), and detailed record keeping (10/10). Other relatively common elements included: rational use of oxygen (7/10), cardiovascular system support (6/10), early nutritional care (8/10), prevention of infection (6/10), laboratory investigation (8/10), and communication with family (6/10). Only one study reported delayed cord clamping (DCC) (1/10).

**Table 3:** Interventions included in the golden hour QI

References	(1)	(2)	(3)	(4)	(5)	(6)	(7)	(8)	(9)	(10)	(11)
Croop et al. [32]	+	+	+	+	+	+	+	+	+	+	+
Peleg et al. [17]	+		+	+	+		+	+	+	+	
Harriman et al. [33]	+		+	+	+	+	+	+	+	+	+
Ashmeade et al. [18]	+		+	+			+		+	+	
Lambeth et al. [34]	+		+	+		+	+	+	+	+	+
Vergales et al. [21]	+		+	+	+		+	+	+	+	+
Reuter et al. [19]	+		+	+		+	+	+	+	+	+
Castrodale et al. [20]	+		+	+	+	+	+		+	+	+
Wallingford et al. [31]	+		+	+	+					+	
Reynolds et al. [35]	+		+	+	+	+				+	

(1) Antenatal counseling and team briefing, (2) delayed cord clamping, (3) prevention of hypothermia, (4) respiratory system support, (5) rational use of oxygen, (6) cardiovascular system support, (7) early nutritional care, (8) prevention of infection, (9) laboratory investigation, (10) keep necessary record, (11) communication with family

**Fig. 2:**
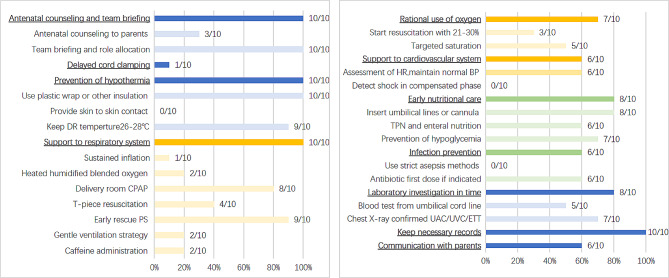
Detailed interventions for each QI bundle element. “n/N” represents the number of included studies/ total number of included studies. DR: delivery room; CPAP: continuous positive airway pressure; PS: pulmonary surfactant; HR: heart rate, BP: blood pressure; TPN: total parenteral nutrition; UAC/UVC: umbilical arterial and venous catheters; ETT: endotracheal tube

Detailed interventions for each element were shown in Fig. 2. Most studies reported two interventions to prevent hypothermia: use of plastic wrap or bag, plastic caps, radiant warmer, thermal mattress, pre-warmed incubators, warm humidified gases (10/10) and maintaining DR temperature at 26–28℃ (9/10). The most common respiratory support included DR continuous positive airway pressure (CPAP) (8/10), T-piece resuscitation (4/10), and early rescue pulmonary surfactant (PS) (9/10). The rational use of oxygen included starting resuscitation with low-oxygen concentrations of 21–30% in preterm neonates (3/10) and targeted saturation (5/10). The most common cardiovascular support was the assessment of heart rate, maintenance of normal perfusion, and blood pressure (6/10). Most studies reported interventions in early nutritional care, including insertion of umbilical lines or cannula (8/10), total parenteral nutrition and enteral nutrition (6/10), starting intravenous fluids infusion, and prevention of hypoglycemia (7/10). The most commonly reported infection control was the use of antibiotics as a first dose if indicated (6/10). Laboratory investigation included two aspects: complete blood count, blood culture, glucose, and arterial blood gas analysis from the central line (5/10) and chest X-ray confirmed the location of umbilical arterial and venous catheters or endotracheal tube (7/10). A detailed record keeping included complete resuscitation record, birth weight, the axillary temperature at admission to nursery, time of surfactant instillation, time of umbilical catheterization, and position of the endotracheal tube, umbilical catheters, and feeding tube. No study reported skin-to-skin contact, detection of shock in compensated phase, and the use of strict asepsis methods.

### Primary outcomes

This review included three primary outcomes (Table 1). The effect on outcomes were summarized in Table S1. The most common primary outcome was admission temperatures (9/10). Five studies reported improvements in admission temperature after QI (*n* = 2308), including improvements in hypothermia (59% vs. 26% vs. 38% for pre-protocol, Phase I, and II, respectively; *p* = 0.001), percentage of reaching target temperature (40% vs. 57%; *p* = 0.001, and 28.3% vs. 49.6%; *p* = 0.002), and other two mean admission temperatures (35.26 vs. 36.26℃; *p* < 0.001 and 36.04 ± 0.81 vs. 36.56 ± 0.82℃ vs. 36.68 ± 0.65 for pre-, initial, and revised QI, respectively; *p* = 0.0001) [17, 18, 20, 32, 35]. One study reported an increasing trend in the percentage of infants admitted with temperature in the normothermic range post-intervention (54.8% vs. 71.3%; *p* = 0.056) [21]. Four studies reported glucose after improvement (*n* = 2052) [17, 18, 20, 32], of which one study reported improvement of hypoglycemia (18% vs. 7% vs. 4% for pre-protocol, phase I, and II, respectively; *p* = 0.012 ) [32], and one reported proportion of glucose > 50 mg/dL increased (72.3% vs. 55.7%; *p* = 0.012) [20], while another study found an increased incidence of hypoglycemia (25.8% vs. 35.1%; *p* = 0.047) [17]; however, no difference was found in subgroups with gestational age ≤ 28 weeks.

### Process outcomes and balancing outcomes

One study did not report any major process outcomes [17] (Table 4). Five studies reported initial infusion time [18, 32–34, 20], and two study found a statistical difference after QI (78.9 ± 43.3 vs. 27.4 ± 12.7 min; *p* < 0.01 and median [IQR], 55 [26] vs. 106 [40]([26]and [40]were [IQR], just a number, not references) minutes; *p* < 0.001) [18, 20]. Three studies reported the time to initiation of antibiotics [32–34] and only one study reported a descending trend in the use of ampicillin and gentamicin [33]. Six studies reported time to surfactant [18, 19, 31, 32, 34, 35], of which one study showed decreased in time (79.8 ± 56.6 vs. 30.8 ± 21.8; *p* < 0.01) [18]. Only one study reported improvement in the stabilization time (median [IQR]: 110 [89–138] vs. 111 [94–135] vs. 92 [74–129] minutes for pre-protocol, Phase I, and II respectively; *p* = 0.004]) [32]. One study reported time to intubation [19] but found no effect. One study reported a decline in time to umbilical line placement [19]. Three studies found no effect in time to NICU admission [18, 19, 21]. Six studies reported major balancing outcomes (Table 4) [17–19, 21, 32, 34]. Two studies found no difference in mechanical ventilation duration [17, 19], and one study reported a decline in PMA on the day of discharge home [21].

**Table 4:** Process outcomes and balancing outcomes included in the golden hour QI

References	Process Outcomes								Balancing Outcomes				
	(1)	(2)	(3)	(4)	(5)	(6)	(7)		(8)	(9)	(10)	(11)	(12)
Croop et al. [32]		#	NR	NR	NR	NR	NR		NR				#
Peleg et al. [17]												#	
Harriman et al. [33]						#	#						
Ashmeade et al. [18]		↓	#				↓						#
Lambeth et al. [34]		NR				NR	NR		NR	NR	NR		
Vergales et al. [21]			#										↓
Reuter et al. [19]	#	#	↑		↓							#	#
Castrodale et al. [20]						↓							
Wallingford et al. [31]		NR		NR									
Reynolds et al. [35]		NR											

**The process outcomes**: (1) time to intubation, (2) time to surfactant, (3) time to NICU admission, (4) time to admission temperature, (5) time to umbilical line placement(confirmation), (6) time to initiation of IV fluids, (7) time to initiation of antibiotics;

**The balancing outcomes**: (8) hyperthermia, (9) insertion-related catheter-associated bloodstream infections, (10) irrational use of antibiotics, (11) mechanical ventilation duration, (12) PMA on the day of discharge home

↓: Decrease; ↑: increase; #: no statistical significance; NR: not report;

NICU: neonatal intensive care unit; PMA: postmenstrual age; IV: intravenous

### Long-term outcomes

**Fig. 3:**
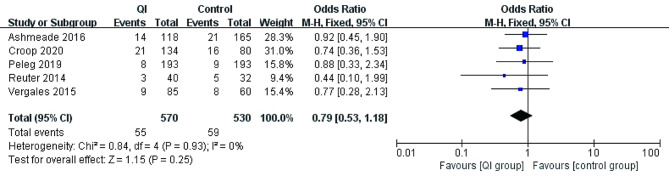
The incidence of severe IVH before and after QI

**Fig. 4:**
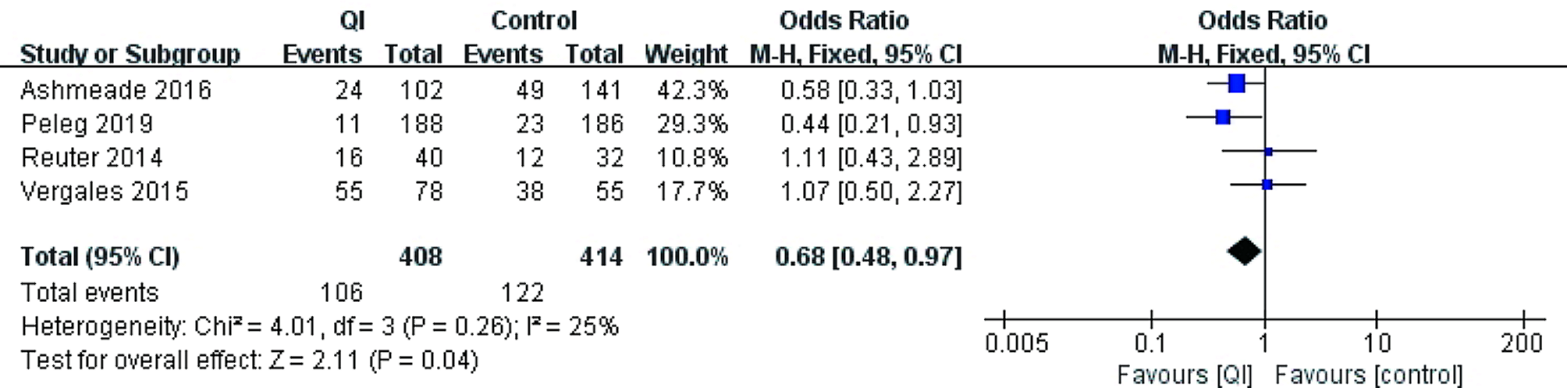
The incidence of BPD before and after QI

**Fig. 5:**
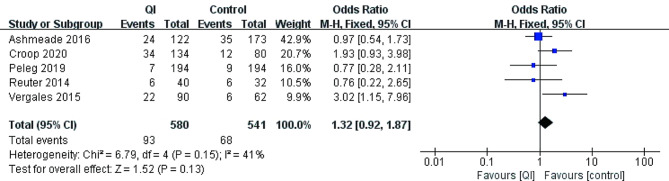
The mortality before and after QI

Long-term outcomes reported in the included studies mostly were comorbidities of prematurity. Three studies did not report any long-term outcomes [20, 33, 34] (Table 1). Five studies reported severe IVH (*n* = 1201) [17–19, 21, 32]. The pooled estimate showed no statistically significant difference after QI: 55/570 (9.6%) vs. 59/530(11.1%) [OR = 0.79, 95% CI 0.53–1.18, *p* = 0.25]. Four studies involving 907 preterm infants showed the incidence of BPD was lower after QI to the controls: 106/408 (26.0%) vs. 122/424 (29.5%) [OR = 0.68, 95% CI 0.48–0.97, *p* = 0.04] [17–19, 21]. Five studies reported mortality (*n* = 1201) [17–19, 21, 32]. The pooled estimate did not show a statistically difference in mortality after QI: 93/580 (16.0%) vs. 68/541(12.6%) [OR = 1.32, 95% CI 0.92–1.87, *p* = 0.13] (Figs. 3, 4 and 5).

One study reported a decline in ROP requiring treatment [18]. One study reported decreased incidence of late-onset sepsis (LOS) [17]. Three studies reported NEC incidence [17–19] while another study reported patent ductus arteriosus (PDA) incidence [19]; however, no statistical difference was found in all studies. Only one study demonstrated improvement in the assessment of clear role definitions in the DR and the organization and efficiency both in the DR and during the NICU admission (*p* < 0.05) [21].

## Discussion

This scoping review including ten studies summarized golden hour QI practices for extremely preterm neonates (GA ≤ 32 weeks and/or BW < 1500 g). The included studies had substantial heterogeneity and deficiencies. Most studies focused on primary and long-term outcomes but lacked detailed definitions of process and balancing measures. Most studies showed improvement in hypothermia, but the range of target temperature varied. Several studies showed improvement in hypoglycemia. However, the QI bundle had no consistent effect on other primary outcomes. A single study found improvement in time to surfactant and time to completion of stabilization respectively. The pooled estimate showed the incidence of BPD decreased after QI, but no statistically significant difference in severe IVH and mortality.

The QI bundle is often complex, multi-component, and customized for specific settings and has also evolved. Valid and reliable critical appraisal tools advance QI intervention impacts by helping stakeholders to identify higher-quality studies [36]. We selected the QI-MQCS checklist to guide the critical appraisal of included studies. Most included studies were of medium quality (80%) and lacked information on the comparator, adherence/fidelity, penetration/reach, sustainability, and spread. To conduct high-quality QI research, attention should be paid to the neglected content domains in addition to the common ones. Other widely used QI assessment methods include Plan-Do-Study-Act (PDSA) cycle, which was first published in 2009 [37], key driver diagrams, Pareto charts, and time-series data analysis [38]. Three included studies used PDSA to evaluate the effectiveness of interventions [18, 32, 34] and one study used the Lean Six Sigma methodology [32]. Four studies used statistical process control charts to analyze process performance [17, 18, 32, 34]. This review found that the application of these methods was published between 2016 and 2020.

There was variability within the bundled elements. The most common elements included antenatal counseling and team briefing, prevention of hypothermia, respiratory system support, and detailed record keeping. All the included studies indicated significant staff education, training, and multidisciplinary teamwork in the QI implementation. Studies have demonstrated the importance of continuous education and training process, team briefing, communication and multidisciplinary collaboration in the success of QI initiatives [39–41]. However, common element such as providing skin-to-skin contact was not reported. A Cochrane review reported that early skin-to-skin contact initiated promote breast-feeding and successful newborn transition to the outside world [42]. Another study found that a dose-dependent relationship appears to exist between skin-to-skin contact and breastfeeding, with longer durations of first contact [43]. Critically, women who have early skin-to-skin contact with their newborns have a lowered risk of postpartum hemorrhage and faster expulsion of the placenta than women who do not experience skin-to-skin contact [42]. Therefore, it is significant to emphasize early skin-to-skin contact in future Golden Hour QI studies. DCC was only reported in one study. However, a survey of Australian and New Zealand Neonatal Network units’ golden hour practices for initial stabilization of very preterm infants showed that DCC was practiced ‘always’ or ‘often’ by 21 units (88%) [44]. A meta-analysis involving 48 studies showed that DCC may reduce the risk of death before discharge (average risk ratio (aRR) 0.73, 95% confidence interval (CI) 0.54–0.98, moderate certainty) compared with early cord clamping [45]. Future studies should focus on the neglected interventions and identify the most important components of individual elements.

Process outcomes that directly contributed to thermoregulation, glycemic control, and time to completion of stabilization were tracked. In our study process measures varied substantially. The measures that closely associated with primary outcomes, such as time to NICU admission, intubation time, central line placement time, could be effective drivers of QI initiatives. Due to a changing airway management in the DR over time, non-invasive support is preferred today. A study about respiratory management protocol in DR, including DCC, in combination with optimized nCPAP with high PEEP levels and less invasive surfactant administration (LISA), showed a lower rate of intubation in DR and were less likely to need mechanical ventilation on day 3 and during the hospital stay after QI, and did not differ in terms of mortality or neonatal morbidity between the two groups [46]. The establishment of a standardized process measurement could help to better implement QI in preterm care and assess the association with outcomes.

Balancing measures are necessary to track in the QI project, which help us spot unintended consequences (good or bad) or issues that can occur as a result of process changes [47]. However, most of the included studies did not clearly define balancing outcomes. It is noteworthy that some balancing measures were ignored, such as hyperthermia, only two studies reported [32, 34], and the latter also reported insertion-related catheter-associated bloodstream infections and antibiotics initiated without an order. On the “golden hour” checklist, “time of initiation of antibiotics” can easily be misinterpreted as all infants need antibiotics regardless of risk factors or physician order. Tracking the duration of mechanical ventilation was to determine if changes in the QI practices affected ventilation time and length of stay. Thus, future studies should comprehensively evaluate the benefits and adverse consequences of QI.

The commonly reported long-term outcomes were BPD (70%), severe IVH (50%) and mortality (50%) in this review. A systemic review involving 22 reports showed that respiratory QI interventions successfully reduced BPD or other key respiratory measures, particularly for infants with BW over 1000 g [32, 34]. Another QI of golden hour management of respiratory distress syndrome in preterm newborns showed that the overall BPD rate decreased from 33.5 to 16.5% [13]. Other long-term outcomes were rarely reported, including ROP, LOS, NEC, PDA, and early blood transfusion, 13 times in total.

The current study summarized the experiences of the implementation and impact of the golden hour QI protocol for extremely preterm infants, which may help inform future studies of higher-quality golden hour QI. To our knowledge, this is the first scoping review to evaluate the effect of the golden hour QI bundle on the outcomes of preterm infants.

However, this systemic review has several limitations. First, quantitative synthesis of primary outcomes was not carried out due to significant heterogeneity across studies, including demographic characteristics, the composition of interventions, implementation methods, definition (e.g. target temperature), interested outcomes, etc. Second, because some of the included trials had small sample sizes and low event rates, selective reporting and publication biases were inevitable. However, current published studies are informative and provide the basis for further studies to improve the golden hour care of preterm infants.

## Conclusion

This scoping review first summarized previous literature on the implementation of the golden hour QI protocol and evaluated its impact on the outcomes of extremely preterm infants (GA ≤ 32 weeks and/or BW < 1500 g). Our study showed that the golden hour QI bundle can improve the short-term and long-term outcomes for extremely preterm infants in the first hour after birth. Most studies showed that the interventions improved hypothermia and hypoglycemia, but the impact on other outcomes was inconsistent. The pooled estimate showed the incidence of BPD decreased after QI, but no statistically significant difference in severe IVH and mortality. The key to the success and maintenance of the Golden Hour QI initiative is a continuous education and training process and the ongoing collaboration of multidisciplinary team members. There was considerable heterogeneity and deficiencies in the included studies, and the variation in impact on outcomes suggests the need to use standardized and validated measures. Future studies are necessary to develop locally appropriate, high-quality, and replicable QI projects.

### Electronic supplementary material

Below is the link to the electronic supplementary material.

**Supplementary Material 1: Supplementary material 1.** Detailed search strategy in PubMed. **Table S1.** Primary outcomes of preterm infants from included studies

## Data Availability

All data generated or analysed during this study are included in this published article and its supplementary information files.

## Abbreviations

Amp: Ampicillin
BP: Blood pressure
BPD: Bronchopulmonary dysplasia
CPAP: Continuous positive airway pressure
DCC: Delayed cord clamping
DR: Delivery room
ELBW: Extremely low birth weight
ETT: Endotracheal tube
GA: Gestational age
Gent: Gentamicin
HR: Heart rate
IQR: Interquartile range
IV: Intravenous
IVH: Intraventricular hemorrhage
LOS: Late-onset sepsis
NEC: Necrotizing enterocolitis
NICU: Neonatal intensive care unit
NR: Not report
PDA: Patent ductus arteriosus
PMA: Postmenstrual age
PS: Pulmonary surfactant
QI: Quality improvement
RCTs: Randomized controlled trials
ROP: Retinopathy of prematurity
SD: Standard deviation
TPN: Total parenteral nutrition
UAC/UVC: Umbilical arterial and venous catheters
VLBW: Very low birth weight

## References

[1] Glass HC, Costarino AT, Stayer SA, Brett CM, Cladis F, Davis PJ. Outcomes for extremely premature infants. Anesth Analg. 2015;120(6):1337–51. 10.1213/ANE.0000000000000705 10.1213/ANE.0000000000000705 PMC4438860 25988638

[2] Vento M, Cheung PY, Aguar M (2009). The first golden minutes of the extremely-low-gestational-age neonate: a gentle approach. NEONATOLOGY.

[3] Wyckoff MH. Initial resuscitation and stabilization of the periviable neonate: the golden-hour approach. Semin Perinatol. 2014;38(1):12–6. 10.1053/j.semperi.2013.07.003 10.1053/j.semperi.2013.07.003 24468564

[4] Finer N, Rich W (2010). Neonatal resuscitation for the preterm infant: evidence versus practice. J PERINATOL.

[5] Katheria A, Rich W, Finer N (2016). Optimizing care of the Preterm Infant starting in the delivery room. Am J Perinatol.

[6] Miller SS, Lee HC, Gould JB. Hypothermia in very low birth weight infants: distribution, risk factors and outcomes. J Perinatol. 2011;31 Suppl 1:S49–56. 10.1038/jp.2010.177 10.1038/jp.2010.177 21448204

[7] Chang HY, Sung YH, Wang SM, Lung HL, Chang JH, Hsu CH (2015). Short- and long-term outcomes in very low Birth Weight infants with Admission Hypothermia. PLoS ONE.

[8] Abiramalatha T, Ramaswamy VV, Bandyopadhyay T, Pullattayil AK, Thanigainathan S, Trevisanuto D (2021). Delivery Room interventions for Hypothermia in Preterm neonates: a systematic review and network Meta-analysis. JAMA PEDIATR.

[9] Dixon KL, Carter B, Harriman T, Doles B, Sitton B, Thompson J (2021). Neonatal thermoregulation: a Golden Hour Protocol Update. Adv Neonatal Care.

[10] Donnellan D, Moore Z, Patton D, O’Connor T, Nugent L. The effect of thermoregulation quality improvement initiatives on the admission temperature of premature/very low birth-weight infants in neonatal intensive care units: A systematic review. J Spec Pediatr Nurs. 2020;25(2): e12286. 10.1111/jspn.12286 10.1111/jspn.12286 31909894

[11] Wang B, Zhang J, Wu YZ, Lu ZH, Wang N, Yu ZB. Reference interval for pulse oxygen saturation in neonates at different altitudes: a systematic review. Front Pediatr. 2021;9:771750. 10.3389/fped.2021.771750 10.3389/fped.2021.771750 PMC8591307 34790638

[12] Chiriboga N, Cortez J, Pena-Ariet A, Makker K, Smotherman C, Gautam S (2019). Successful implementation of an intracranial hemorrhage (ICH) bundle in reducing severe ICH: a quality improvement project. J PERINATOL.

[13] Dylag AM, Tulloch J, Paul KE, Meyers JM. A quality improvement initiative to reduce bronchopulmonary dysplasia in a level 4 NICU-golden hour management of respiratory distress syndrome in preterm newborns. Children (Basel). 2021;8(4). 10.3390/children8040301 10.3390/children8040301 PMC8071250 33920871

[14] Conway-Orgel M. Management of hypotension in the very low-birth-weight infant during the golden hour. Adv Neonatal Care. 2010;10(5):241–5, 246-7. 10.1097/ANC.0b013e3181f0891c 10.1097/ANC.0b013e3181f0891c 20838073

[15] Mitra S, Disher T, Pichler G, D’Souza B, Mccord H, Chayapathi V et al. Delivery room interventions to prevent bronchopulmonary dysplasia in preterm infants: a protocol for a systematic review and network meta-analysis. BMJ Open. 2019;9(8):e28066. 10.1136/bmjopen-2018-028066 10.1136/bmjopen-2018-028066 PMC6701811 31427322

[16] Healy H, Croonen L, Onland W, van Kaam AH, Gupta M. A systematic review of reports of quality improvement for bronchopulmonary dysplasia. Semin Fetal Neonatal Med. 2021;26(1):101201. 10.1016/j.siny.2021.101201 10.1016/j.siny.2021.101201 33563565

[17] Peleg B, Globus O, Granot M, Leibovitch L, Mazkereth R, Eisen I et al. Golden hour quality improvement intervention and short-term outcome among preterm infants. J Perinatol. 2019;39(3):387–92. 10.1038/s41372-018-0254-0 10.1038/s41372-018-0254-0 30341403

[18] Ashmeade TL, Haubner L, Collins S, Miladinovic B, Fugate K. Outcomes of a neonatal golden hour implementation project. Am J Med Qual. 2016;31(1):73–80. 10.1177/1062860614548888 10.1177/1062860614548888 25194002

[19] Reuter S, Messier S, Steven D (2014). The neonatal Golden Hour–intervention to improve quality of care of the extremely low birth weight infant. S D Med.

[20] Castrodale V, Rinehart S. The golden hour: improving the stabilization of the very low birth-weight infant. Adv Neonatal Care. 2014;14(1):9–14, 15–6. 10.1097/ANC.0b013e31828d0289 10.1097/ANC.0b013e31828d0289 24472882

[21] Vergales BD, Dwyer EJ, Wilson SM, Nicholson EA, Nauman RC, Jin L et al. NASCAR pit-stop model improves delivery room and admission efficiency and outcomes for infants < 27 weeks’ gestation. Resuscitation. 2015;92:7–13. 10.1016/j.resuscitation.2015.03.022 10.1016/j.resuscitation.2015.03.022 25891960

[22] Lamary M, Bertoni CB, Schwabenbauer K, Ibrahim J. Neonatal golden hour: a review of current best practices and available evidence. Curr Opin Pediatr. 2023;35(2):209–17. 10.1097/MOP.0000000000001224 10.1097/MOP.0000000000001224 36722754

[23] Tricco AC, Lillie E, Zarin W, O’Brien KK, Colquhoun H, Levac D et al. PRISMA extension for scoping reviews (PRISMA-ScR): checklist and explanation. Ann Intern Med. 2018;169(7):467–73. 10.7326/M18-0850 10.7326/M18-0850 30178033

[24] Shivananda S, Gupta S, Thomas S, Babb L, Meyer CL, Symington A et al. Impact of a dedicated neonatal stabilization room and process changes on stabilization time. J Perinatol. 2017;37(2):162-7. 10.1038/jp.2016.205 10.1038/jp.2016.205 27831550

[25] Lapcharoensap W, Bennett MV, Powers RJ, Finer NN, Halamek LP, Gould JB et al. Effects of delivery room quality improvement on premature infant outcomes. J Perinatol. 2017;37(4):349–54. 10.1038/jp.2016.237 10.1038/jp.2016.237 28005062

[26] Persaud A, Hodgson K, Bailey J, Owen LS. The neonatal ‘golden hour’: an audit of practice in infants below 30 weeks gestational age. J Paediatr Child H. 2018;54:101. 10.1111/jpc.13882_272

[27] Habib AS, Linn Leukart R, Brown A, Bartman T. Golden hour for extremely premature infants: improving time to normothermia and administration of ivf and antibiotics. Pediatrics. 2018;141(1). 10.1542/peds.141.1-MeetingAbstract.114

[28] Ardern J, McKinlay C, Vandal A, Hayward B. The golden hour: team lanyards improve nurse confidence and care coordination during neonatal admission. J Paediatr Child H. 2020;56(SUPPL 1):58–9. 10.1111/jpc.14832

[29] Ibrahim K, Mulla S. Neonatal super 60 project. Arch Dis Child. 2022;107:A131–2. 10.1136/archdischild-2022-rcpch.213

[30] Hemingway M, Raju M, Vora N, Raju V, Mallett LH, Govande V. Improving delivery room and admission efficiency and outcomes for infants < 32 weeks: ELGAN+ (extremely low gestational age neonate). J Neonatal Perinatal Med. 2022. 10.3233/NPM-210881 10.3233/NPM-210881 36591661

[31] Wallingford B, Rubarth L, Abbott A, Miers LJ. Implementation and evaluation of golden hour practices in infants younger than 33 weeks’ gestation. Newborn & Infant Nursing Reviews. 2012;12(2).

[32] Croop S, Thoyre SM, Aliaga S, McCaffrey MJ, Peter-Wohl S. The golden hour: a quality improvement initiative for extremely premature infants in the neonatal intensive care unit. J Perinatol. 2020;40(3):530-9. 10.1038/s41372-019-0545-0 10.1038/s41372-019-0545-0 PMC7222905 31712659

[33] Harriman TL, Carter B, Dail RB, Stowell KE, Zukowsky K. Golden hour protocol for preterm infants: a quality improvement project. Adv Neonatal Care. 2018;18(6):462–70. 10.1097/ANC.0000000000000554 10.1097/ANC.0000000000000554 30212389

[34] Lambeth TM, Rojas MA, Holmes AP, Dail RB. First golden hour of life: a quality improvement initiative. Adv Neonatal Care. 2016;16(4):264–72. 10.1097/ANC.0000000000000306 10.1097/ANC.0000000000000306 27391563

[35] Reynolds RD, Pilcher J, Ring A, Johnson R, McKinley P. The golden hour: care of the LBW infant during the first hour of life one unit’s experience. Neonatal Netw. 2009;28(4):211–9, 255–8. 10.1891/0730-0832.28.4.211 10.1891/0730-0832.28.4.211 19592362

[36] Hempel S, Shekelle PG, Liu JL, Sherwood DM, Foy R, Lim YW et al. Development of the quality improvement minimum quality criteria set (QI-MQCS): a tool for critical appraisal of quality improvement intervention publications. BMJ Qual Saf. 2015;24(12):796–804. 10.1136/bmjqs-2014-003151 10.1136/bmjqs-2014-003151 PMC4680162 26311020

[37] Sheldon KA, Seoane-Vazquez E, Szeinbach SL, Tubbs C. Using the plan-do-study-act model to convert to a new insulin delivery system. Am J Health Syst Pharm. 2009;66(12):1074–5. 10.2146/ajhp080185 10.2146/ajhp080185 19498116

[38] Picarillo AP. Introduction to quality improvement tools for the clinician. J Perinatol. 2018;38(7):929–35. 10.1038/s41372-018-0100-4 10.1038/s41372-018-0100-4 29795322

[39] Kamath-Rayne BD, Josyula S, Rule A, Vasquez JC. Improvements in the delivery of resuscitation and newborn care after helping babies breathe training. J Perinatol. 2017;37(10):1153–60. 10.1038/jp.2017.110 10.1038/jp.2017.110 28726790

[40] Sachan R, Srivastava H, Srivastava S, Behera S, Agrawal P, Gomber S. Use of point of care quality improvement methodology to improve newborn care, immediately after birth, at a tertiary care teaching hospital, in a resource constraint setting. BMJ Open Qual. 2021;10(Suppl 1). 10.1136/bmjoq-2021-001445 10.1136/bmjoq-2021-001445 PMC8336133 34344737

[41] Ohlinger J, Kantak A, Lavin JJ, Fofah O, Hagen E, Suresh G et al. Evaluation and development of potentially better practices for perinatal and neonatal communication and collaboration. Pediatrics. 2006;118 Suppl 2:S147–52. 10.1542/peds.2006-0913L 10.1542/peds.2006-0913L 17079617

[42] Moore ER, Bergman N, Anderson GC, Medley N. Early skin-to-skin contact for mothers and their healthy newborn infants. Cochrane Database Syst Rev. 2016;11(11):D3519. 10.1002/14651858.CD003519.pub4 10.1002/14651858.CD003519.pub4 PMC6464366 27885658

[43] Redshaw M, Hennegan J, Kruske S. Holding the baby: early mother-infant contact after childbirth and outcomes. Midwifery. 2014;30(5):e177–87. 10.1016/j.midw.2014.02.003 10.1016/j.midw.2014.02.003 24680108

[44] Hodgson KA, Owen LS, Lui K, Shah V. Neonatal golden hour: a survey of Australian and New Zealand neonatal network units’ early stabilisation practices for very preterm infants. J Paediatr Child Health. 2021;57(7):990–7. 10.1111/jpc.15360 10.1111/jpc.15360 33543835

[45] Rabe H, Gyte GM, Díaz-Rossello JL, Duley L. Effect of timing of umbilical cord clamping and other strategies to influence placental transfusion at preterm birth on maternal and infant outcomes. Cochrane Database Syst Rev. 2019;9(9):D3248. 10.1002/14651858.CD003248.pub4 10.1002/14651858.CD003248.pub4 PMC6748404 31529790

[46] Templin L, Grosse C, Andres V, Robert CD, Fayol L, Simeoni U et al. A quality improvement initiative to reduce the need for mechanical ventilation in extremely low gestational age neonates. Am J Perinatol. 2017;34(8):759–64. 10.1055/s-0037-1598106 10.1055/s-0037-1598106 28142154

[47] Shah A. Using data for improvement. BMJ. 2019;364:l189. 10.1136/bmj.1189 10.1136/bmj.l189 PMC6376414 30770353

