# Supplementary material for: Quality improvement in the golden hour for premature infants: a scoping review
Source: BMC Pediatr. 2024 Feb 1;24:88. doi: 10.1186/s12887-024-04558-9 (PMC10832117; doi:10.1186/s12887-024-04558-9)
Supplement: Supplementary file 1 — Supplementary Material 1: Supplementary material 1. Detailed search strategy in PubMed. Table S1. Primary outcomes of preterm infants from included studies [file 12887_2024_4558_MOESM1_ESM.docx]

**Supplementary material 1.** Detailed search strategy in PubMed.

| (“infant, premature” [MeSH Terms] OR “premature” [Title/Abstract] OR “prematurity” [Title/Abstract] OR “preterm” [Title/Abstract] OR “pre term” [Title/Abstract] OR “low birth weight” [Title/Abstract] OR “low birthweight” [Title/Abstract] OR “very low birth weight” [Title/Abstract] OR “extremely low birth weight” [Title/Abstract] OR “LBW” [Title/Abstract] OR “VLBW” [Title/Abstract] OR “ELBW” [Title/Abstract] OR (“infan*” [Title/Abstract] OR “neonat*” [Title/Abstract]) OR (“infant, newborn” [MeSH Terms] OR “newborn*” [Title/Abstract] OR “new born” [Title/Abstract] OR “new borns” [Title/Abstract] OR “newly born” [Title/Abstract] OR “baby*” [Title/Abstract] OR “babies*” [Title/Abstract])) AND (“golden” [All Fields] AND (“hour” [All Fields] OR (“sixty” [All Fields] AND (“minute” [All Fields] OR “minutes” [All Fields])) OR (“60” [All Fields] AND (“minute” [All Fields] OR “minutes” [All Fields])))) |
| --- |

Retrieval time was from the inception of the database to April 03, 2023.

**Table S1.** Primary outcomes of preterm infants from included studies.

| **Outcomes** | **Studies** | **Statistic** | **QI group** | **Control group** | **P value** |
| --- | --- | --- | --- | --- | --- |
| Admission  temperatures | Ashmeade et al.[18] | %, n/N (≥36.4℃) | 56.5% (69/122) | 40% (68/170) | 0.005 |
|  | Croop et al. [32] | %, n/N (36.5–37.5 °C) | 67.1% (90/134) | 42.5% (34/80) | 0.001 |
|  | Castrodale et al.[20] | %, n/N (36.0–37.4℃) | 49.6% (53/119) | 28.3% 30/106) | 0.002 |
|  | Peleg et al.[17] | Mean (℃) | 36.26 | 35.26 | <0.001 |
|  | Peleg et al. [17]  (≤28week） | Mean (℃) | 36.25 | 34.93 | <0.001 |
|  | Reynolds et al.[35] | Mean (SD) (℃) | 36.56(0.82),  36.68(0.65) * | 36.04(0.81) | 0.0001/  0.0001 |
|  | Harriman et al.[33] | %, n/N (>36.5°C) | 100% (7/7) | 76.4% (13/17) | 0.28 |
|  | Vergales et al.[21] | %, n/N (36.0–37.5℃) | 71.2% (62/87) | 54.8% (34/62) | 0.056 |
| Glucose | Croop et al.32] | %, n/N (<45 mg/dL) | 5.2% (7/134) | 17.5% (14/80) | 0.012 |
|  | Peleg et al.[17] | %, n/N (<45 mg/dL) | 35.1%（68/194） | 25.8%（50/194） | 0.047 |
|  | Peleg et al. [17]  (≤28week） | %, n/N (<45 mg/dL) | 28.5% (14/49) | 16.3% (8/49) | 0.151 |
|  | Castrodale et al.[20] | %, n/N (>50 mg/dL) | 72.3% (86/119) | 55.7% (59/106) | 0.012 |
|  | Ashmeade et al.[18] | Mean(SD)（mg/dl） | 72(29) | 67(30) | 0.143 |
| Time to initiation  of IV fluids | Ashmeade et al.[18] | Mean(SD)（min） | 27.4 (12.7) | 78.9 (43.3) | <0.01 |
|  | Harriman et al.[30] | Mean (SD)(min) | 44.1 (10.3) | 67.1 (62.5) | 0.35 |
|  | Castrodale et al.[20] | Median (IQR)(min) | 55 (26) | 106 (40) | <0.001 |
| Time to initiation  of antibiotics | Harriman et al.[33] | Mean (SD)(min)  (Amp) | 96.6（29） | 111.2（72.2） | 0.62 |
|  | Harriman et al.[33] | Mean (SD)(min)  (Gent) | 127.1（19.8） | 154.1（76.4） | 0.25 |
| Time to surfactant | Ashmeade et al.[18] | Mean (SD)(min) | 30.8（21.8） | 79.8(56.6） | <0.01 |
|  | Reuter et al.[19] | Median (IQR)(min) | 16[13,25] | 20[13,34] | 0.379 |
| Time to completion  of stabilization | Croop et al.[32] | Median (IQR)(min） | 111[94,135],  92[74,129] * | 110 [89,138] | 0.004 |
| Mean arterial  blood pressure | Vergales et al.[21] | %, n/N | 76.1% (67/88) | 57.4% (35/61) | 0.02 |

Amp, ampicillin; Gent, gentamicin; SD, standard deviation; IQR, interquartile range.

* represents two stages (phase I and phase II) of QI project respectively.
